# How do people with knee osteoarthritis perceive and manage flares? A qualitative study

**DOI:** 10.3399/BJGPO.2021.0086

**Published:** 2022-03-09

**Authors:** Emma Parry, Lisa Dikomitis, George Peat, Carolyn A Chew-Graham

**Affiliations:** 1 School of Medicine, Keele University, Keele, UK

**Keywords:** osteoarthritis, knee arthritis, musculoskeletal, flares, primary health care, qualitative research

## Abstract

**Background:**

Acute flares in people with osteoarthritis (OA) are poorly understood. There is uncertainty around the nature of flares, their impact, and how these are managed.

**Aim:**

To explore understandings and experiences of flares in people with knee OA, and to describe self-management and help-seeking strategies.

**Design & setting:**

Qualitative interview study of people with knee OA in England.

**Method:**

Semi-structured interviews were undertaken with 15 people with knee OA. Thematic analysis was applied using constant comparison methods.

**Results:**

The following four main themes were identified: experiencing pain; consequences of acute pain; predicting and avoiding acute pain; and response to acute pain. People with OA described minor episodes that were frequent, fleeting, occurred during everyday activity, had minimal impact, and were generally predictable. This contrasted with severe episodes that were infrequent, had greater impact, and were less likely to be predictable. The latter generally led to feelings of low confidence, vulnerability, and of being a burden. The term ‘flare’ was often used to describe the severe events but this was applied inconsistently and some would describe a flare as any increase in pain. Participants used numerous self-management strategies but tended to seek help when these had been exhausted, their symptoms led to emotional distress, disturbed sleep, or pain experience worse than usual. Previous experiences shaped whether people sought help and who they sought help from.

**Conclusion:**

Severe episodes of pain are likely to be synonymous with flares. Developing a common language about flares will allow a shared understanding of these events, early identification, and appropriate management.

## How this fits in

‘Flares’ are a poorly understood concept in OA. People with knee OA define flares inconsistently, but commonly these would represent severe episodes of pain that are infrequent, sustained, and sometimes unpredictable, which can lead to emotional exhaustion and impact on ability to undertake usual activities and help-seeking. Understanding the reasons for help-seeking and educating people with OA about flares will enable prompt identification and management of these events by primary care clinicians.

## Introduction

Flares or exacerbations in long-term conditions, for example, in rheumatoid arthritis,^1^ gout,^2^ chronic obstructive pulmonary disease,^3^ and asthma^4^ are well documented. There is guidance on how to identify flares and evidence-based recommendations for management in some conditions. In contrast, in OA, ‘flares’ are only starting to receive attention. Recent studies have highlighted the inconsistent way in which OA flares are defined^5^ and have focused on identifying triggers for flares, which has included knee buckling and injury,^6^previous hip injury and giving way.^7^ The variable nature of pain in OA has been well described from focus group^8–10^ and daily diary studies.^11,12^ Pain is a key feature in OA and leads to increased healthcare usage.^13^

In the UK, it is estimated that 8.75 million people have OA,^14^ and that nearly 1 million consult a GP each year with OA-related symptoms in England.^15^ The experiences of people living with chronic OA are well documented,^16^ but less is known about experiences of acute events or flares.

The concept of acute episodes of pain in OA has been described by Hawker *et al* as being intermittent and predictable early on in the disease course, but becoming unpredictable and more distressing as OA progresses.^10^ These painful episodes of OA have contributed to work and productivity loss.^17^ Understanding more about what makes these episodes distressing, potential triggers and predictability will improve episode management. Recognition of flares as part of the disease course in OA will help improve communication between clinicians, researchers, and those experiencing OA. Early recognition may contribute to a modified symptom trajectory, which could mitigate long-term adverse outcomes.

Murphy *et al* conducted interviews with people with OA to explore their perception of flares.^18^ Their definition of flare (*’* *inadequate pain relief for an episode of intense pain that is usually brought on by too much activity’*) encompassed the experience of participants in only a half of cases. The majority of participants described their flares in terms of pain quality (for example, sharp) compared with intensity. The events that were reported were largely short-lived, lasting up to 15 minutes, which contrasts with estimates from other studies where flares were estimated to last a median of 8 days (range 2–30 days).^11^ It remains unclear whether there is a distinction between the short-lived and longer episodes of pain, what the term ‘flare’ captures, and whether this is important to patients. Understanding the impact and importance of these different episodes of pain, including duration, experience, potential triggers, and management, will improve knowledge of flares and provide tailored advice for health professionals managing people with OA.

This study explored the meaning of flares in people with knee OA, how they differentiate between different severities of acute events or flares, impact of flares, their predictability and whether this changes over time, and management strategies employed.

## Method

### Study design and setting

Semi-structured interviews were conducted face-to-face with people with knee OA. A Patient Advisory Group (PAG) was involved in development of the study protocol, public-facing documents, and interpretation of study findings. The knee was investigated as it is the most common joint affected by OA;^19^ diagnostic criteria included those aged ≥45 years^20^ and clinical signs that were easy to identify.^21^

### Sampling and recruitment

People with OA aged ≥45 years were identified through GP records if they had a Read-coded problem in the past 2 years for knee OA or knee pain and/or arthralgia, and who were not in a vulnerable group as judged by their GP before the mailout. Exclusion criteria included those with a previous diagnosis of the following: inflammatory disease (rheumatoid arthritis and polymyalgia rheumatica), crystal disease (gout), spondyloarthropathy, fibromyalgia, or previous total knee replacement at GP list screening. Potential study participants were sent an information pack by their GP practice, which included a participant information sheet and consent to contact form.

Potential study participants were asked the following question: *In the last 12 months have you had an increase of your knee pain, that is times when your knee pain is worse than normal which may have stopped you from doing your normal activities or meant you had to increase your pain medication?* In total 47 potentially eligble participants returned the reply slip indicating a willingness to take part in the study and reported a recent knee flare-up in the past 12 months by answering ‘yes’ to the question above. These were purposively sampled based on age and sex. This was to ensure the participants included in the study contributed data reporting a range of views and experiences.^22^ This question was developed by the research team and was based on previous work.^5,11^ Written consent was obtained before each interview.

### Data collection

Interviews were conducted in the participants’ own homes by the first author, who used a topic guide informed by the literature and refined after PAG engagement (see Supplementary Box S1) to direct the conversation. Topics included participants’ experiences of knee OA, descriptions of flares, management, and help-seeking strategies. The topic guide was modified iteratively as data collection and analysis was ongoing.

During the interview participants were shown example graphs of pain intensity over time, which were adapted from Stone *et al*, to give participants an idea of different types of symptom courses.^23^ Participants were then invited to draw a diagram of their disease course over the previous 6 months and to indicate flare-ups or to identify which of the example graphs best described their pain experiences (see Supplementary Box S2). This helped to facilitate in-depth discussion.

The first author is a practising GP with training in qualitative methods. The participants were not explicitly told that the interviewer was a GP and the first author’s professional status was not discussed in the interviews.

Interviews were transcribed verbatim and pseudonymised using a unique participant identification number. Recruitment and interviews continued until data saturation was achieved after 15 interviews.^24,25^

### Data analysis

Data analysis was ongoing and iterative. The data were analysed using constant comparison methods,^26^ where the researcher interacts and is actively involved in the data and the emerging analysis, making comparisons at each stage. The analysis was undertaken separately by the first author and two researchers, followed by team discussions at each of the coding stages in order to arrive at an agreed coding framework. Emerging themes were discussed with the PAG at the end of the analysis stage, once data generation was complete.

Leventhal’s Self-Regulatory Model (SRM) was used towards the end of the analysis once the overarching themes had been identified, and was used as a framework to aid understanding and organisation of the themes. The SRM provides a framework for understanding people’s attitudes, feelings, and actions about their own health and is a helpful model for understanding these behaviours.^27^ The SRM contains the following five key elements: how people identify with their condition; beliefs on duration; impact of their condition; understanding of causes; and beliefs on whether their symptoms can be cured or controlled.

## Results

The demographics of the 15 participants interviewed are presented in Table 1. All participants were White British.

**Table 1.** Characteristics of interviewees

ID	Sex	Age, years	Reported duration of knee pain, years	Living status
01	M	51	3	Lives with partner
02	M	78	50	Lives with spouse
03	F	66	1	Lives alone
04	F	81	11	Lives with spouse
05	M	59	20	Lives with spouse
06	F	68	7	Lives with spouse
07	M	83	3	Lives with spouse
08	M	66	30	Lives with spouse
09	M	64	≥2	Lives alone
10	F	69	6	Lives with spouse
11	F	70	5	Lives with spouse
12	M	81	60	Lives alone
13	F	78	4–5	Lives with spouse
14	M	85	6	Lives with spouse
15	F	85	1	Lives with daughter

F = female. M = male.

Participants did not always make a clear and consistent distinction between ‘flares’ and other episodes of acute pain. In order to ensure descriptions of flares were kept true to the data and not over-interpreted they are discussed in the context of episodes of acute pain.

For the purposes of the results, the data will be presented within the following four main themes: 1) experiencing pain; 2) consequences of acute pain; 3) predicting and avoiding acute pain; and 4) responses to acute pain. The authors have highlighted which parts of the SRM each theme links to.

### Experiencing pain (*link to timeline and identity of the SRM*)

When describing their pain experience participants did not always use the term ‘flare’ but they used different ways of illustrating times when their pain worsened. Here is how one study participant explained the pain experience:

*’To me it suggests something that erm it sort of comes out of the blue and just sort of suddenly attacks the knee sort of thing you know, yeah, yeah but I’ve never said I’ve had a flare-up of my knee, it’s not a term I would use truly you know.’* (Participant [P]08, male [M], aged 66 years)

The term ‘flare’, which some people used to describe certain types of acute episodes of pain, meant different things to different people. For some it meant a pain that was a longer duration to usual pain, was more severe than usual pain, and impacted on ability to carry out usual activities. However, for others ‘flare’ encompassed any increase in pain and for any duration:

*’When it becomes unbearable erm, but I mean sometimes it’s just for a short while and other times for a day even erm or could be longer but it depends.’* (P07, M, aged 83 years)

Acute episodes of pain were identified and described in a number of ways; for example, change in pain descriptors, change in intensity and magnitude of pain, the speed of onset, their duration, and frequency.

Participants defined acute events as an increase in pain intensity, magnitude of pain, and a shift in pain quality; for example, *‘sharp’* (P01, M, aged 51 years) , *‘stabbing’* (P09, M, aged 64 years), *‘red-hot poker’* (P15, F, aged 85 years), and *‘tonnes of knives’* (P03, F, aged 66 years). This maps onto identity of the SRM. Associated symptoms included: swelling (variable and sometimes present before the pain); stiffness (particularly after being sedentary); and interference with sleep owing to night-time pain.

**Figure 1.**
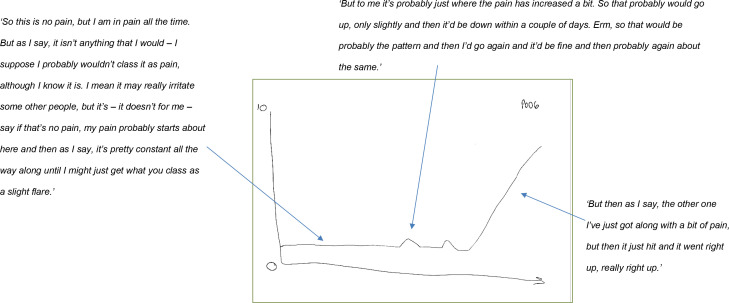
Illustration of pain variability over the previous 6 months on a 0–10 numerical scale with annotation (P06, F, aged 68 years)

Participants highlighted the contrast between minor painful episodes and more severe episodes. Minor painful episodes were frequent, short-lived, tended to occur with daily activity, had minimal impact, and were more likely to be referred to as *‘just one of those days’* (P06, F, aged 68 years). This contrasted with episodes that were described as big and were less frequent but had more of an impact, were sustained, and were recalled with clarity. In Figure 1, P06 described a low-level background pain the majority of the time, which was interrupted by minor increases in pain that usually lasted for short periods of time. However, she described experiencing an unexpected sudden increase in pain intensity that was severe and markedly above usual increases in pain intensity.

The timeline of these acute events, described using the SRM, encompassed their speed of onset and frequency. Onset was sudden or gradual; however, this could be variable and changed over time. Duration and frequency were also variable, with events lasting from minutes to up to 2 months, and occurring daily to every 2 months or so.

### Consequences of acute pain (*link to consequence of the SRM*)

The impact of acute events maps onto consequence of the SRM. Participants shared their frustration when these painful episodes impacted on their ability to participate in certain activities. Interviewees explained that this was more important than the severity or duration of the symptoms.

A significant aspect of mitigating the effect of increases in symptoms was the ability to carry on with usual activities:

*’… it’s not so much the pain that I can’t cope with, it’s the inability to, that I can’t do things that I find is more debilitating than the actual pain.’* (P04, female [F], aged 81 years)

Acute episodes also influenced how much participants could engage in social activities; for example, meeting up with friends and family. P10 (F, aged 69 years) described how she felt she was a burden when she was meeting friends and family. For instance, when she would have to stop regularly during shopping.

Acute episodes could make people with OA feel vulnerable. They described feeling anxious and fearful about future increases in pain and its consequences. This seemed to impact on the participants’ feelings of independence; for example, some would not go shopping alone.

*’* *When I’m walking I feel very insecure, I don’t feel safe, very vulnerable, I keep thinking my knee’s going to give way and I’m going to fall. Er, I’m alright if my husband’s with me and I can hold his arm, or in the supermarket and I can hold a trolley or if I’ve got a walking stick that gives me confidence. But without that just walking from the back door to my washing line, I feel very vulnerable, very insecure yes.’* (P04, F, aged 81 years)

The impact of these episodes was kept to a minimum if they did not interfere with ability to undertake activities, even if this was at a slower pace:

*‘*[On change in frequency over time] *No, no they are more regular. But as I say, I can honestly say they don’t really — I wouldn’t say bother me, but I know that they’re there and yes I have the pain, but they don’t stop me doing anything other than if I have to take it a bit steadier up the stairs or an incline.’* (P06, F, aged 68 years)

There was a strong sense of stoicism, *‘I kind of manage different ways because I just feel you need to get on with it ... So I, I just do it. If, if I can't do it, I don't do it … ’* (P11, F, aged 70 years), despite the pain, and an acceptance for some that acute increases in pain were part of their OA experience. People with OA did not want pain to interfere with their ability to participate in activities. Continuing with daily pursuits despite pain meant activities needed to be adjusted, often took longer, and required the person to direct more thought towards what actions might bring on or prevent increases in their pain.

*’Erm, to me it’s just normal I think now, just to have a flare-up. I get a pain and erm, yeah there maybe the odd day when it’s only minimal pain, not much, but there’s never not pain. Cos whenever I do anything, it hurts, but you have to do things. You know, I can’t not do it.’* (P10, F, aged 69 years)

Many participants mentioned making adaptations to minimise the effect of acute pain; for example, climbing the stairs using their *‘good leg’* (P06, F, aged 68 years) first, or being more observant about foot placement when out walking. These adaptations, however, were perceived as being intrusive and participants seemed to be resentful about having to make them:

*’* *I just have to be very, very careful, watch where I’m walking. I hate having to keep looking down to see where I’m going and it’s tiring, like last week. Happened to look up and didn’t notice this paving stone sticking up and just caught it with my toe.’* (P14, M, aged 85 years)

The overall impact of acute episodes was variable, sometimes people could continue activities despite the pain, while other times it would cause them to stop activity:

*’It’s when I’m doing stuff, as soon as I’m doing stuff I do get a pain and I just have to grit my teeth and go through it and sometimes it’s that bad I have to stop and go and sit down and erm just make my cup of tea and I’ll wait till it eases off, then I can do a little bit more.’* (P10, F, aged 69 years)

### Predicting and avoiding acute pain (*link to cause and consequence of the SRM*)

Predicting and avoiding acute pain was understood using cause and consequence of the SRM. Pain that was unpredictable in terms of its severity, onset, and duration was associated with distress, and seemed to have a greater impact on mental wellbeing and quality of life in terms of having to cancel planned activities:

*’Yeah, it’s quite depressing cos if I’m planning to do something and then I sort of can’t because it’s started to hurt and I know it’s not gonna go away quick*.*’* (P10, F, aged 69 years)

Predictable pain was usually a consequence of everyday, low-bearing activities; for example, walking or climbing stairs. Predictability was important as it enabled activity avoidance, adjustment, and the ability to plan ahead in order to participate in organised activities:

*’I’ve been in pain since June of last year with it. And there are days when I don’t have as much pain as normal, erm but I rested up on Monday because I was going out yesterday and it was a lot of walking. So this morning it is playing me up a bit.’* (P03, F, aged 66 years)

Participants modified their behaviour with the hope of avoiding acute increases in symptoms; for example, P03 indicated above that she rests the day before an activity. However, the variable nature of the predictability of pain meant they still experienced acute events without warning, which often caused distress owing to the inability to identify an underlying cause.

A common thread in participants’ narratives was the sense of guilt they felt when they had deliberately overdone things or embarked on an activity that resulted in acute pain:

*’… so I’ve got no pain and then if I do something, it shoots up, I get me pain and then it takes X amount of days for it to go back down again and then I haven’t got any pain again until I do something similar again which you think, you stupid person, why do you keep doing things but you’ve got to be active haven’t you, you’ve got to do something.’* (P12, M, aged 81 years)

### Responses to acute pain (*link to control of the SRM*)

Participants’ initial responses to acute OA pain were stopping current activity and employing self-management strategies such as taking medication (over-the-counter or prescription only) and/or ignoring the symptoms. A number of people signalled the active role they took in managing their symptoms and this linked to the ‘control’ part of the SRM.

In the short to medium term, people described making adaptations, avoidance of certain activities, and help-seeking from peers or health professionals. Adaptations included use of mobility aids, walking sticks, and alterations to prevent and minimise impact of flares.

People sought help from healthcare professionals when their pain experience did not match their illness perception. This included sleep disturbance, reaching emotional limitations, exhausting self-management options, and pain experience worse than normal; for example, increased severity, and sustained and longer duration to usual worsening of their symptoms:

*’* *…* *when I had the flare up, as you call it, last year, I mean I tried everything on my knee. I tried the heat pad, I sat one day, nearly all day with a heat pad on, but it just did nothing at all. I had a hot-water bottle on it, you just try anything in the end just to get some relief. Erm, but the night I just used to dread* *…* *I was just* *—* *I was at my wits* *’* *end with it in the end. And I’ve never got to that point that last year was the first time where I’d really got to that point* *…* *But it was* *—* *I mean I can stand a fair bit of pain, but I think I got to the point with it because I was having no sleep, I was just worn out with it in the end* *…* *’* (P06, F, aged 68 years)

Some participants described a sense of futility about seeking help owing to previous experiences. For example, being told not much could be done or dissatisfaction with previous management.

Peers were another source of noteworthy advice shaping theories on relative importance and giving opinions on certain management strategies, from home remedies to surgery:

*’I mean a lot of people have knee surgery don’t they? I’ve got my golfing colleagues they’ve all had some say yes some have had two done over the years. And wish they hadn’t have done and it’s not what they expected it to be.’* (P02, M, aged 78 years)

## Discussion

### Summary

People with OA described two different types of events. Minor, fleeting episodes of pain that had minimal impact and were usually triggered by everyday activity such as walking and stairs. These contrasted with descriptions of more severe and rarer episodes of pain that had a greater impact and were more sustained. Although some people referred to only the latter as a ‘flare’, the term was used inconsistently as some described any increase in pain as a ‘flare’. The term ‘flare’ therefore seems to have no clear fixed meaning to people with OA.

Acute episodes of pain, particularly those that were unpredictable, led to a loss of confidence, feelings of vulnerability, and concerns of being a burden to others.

Participants described reasons for help-seeking from health professionals and this tended to be during more severe episodes of pain, when associated with emotional exhaustion, interference with sleep, and pain experience different to the usual increase in symptoms.

### Strengths and limitations

The study had several strengths. In total, 47 participants responded to the mailing invitation and were eligible to take part in the study. This enabled purposive sampling to take place based on age and sex. Data were collected until data saturation was achieved.^24,25^

A PAG was involved at all stages of the study, including discussion of the data and interpretation. The analysis was conducted by a team of clinicians and researchers from different backgrounds, which improved the trustworthiness of the analysis.^28^

The pain graphs drawn or identified by participants were a helpful tool in understanding their pain experience and facilitated an in-depth discussion. The majority of participants preferred to draw their own graphs rather than use the diagrams.

A key limitation of this study was that all of those interviewed identified as White British and lived in the West Midlands area of England.

### Comparison with existing literature

Intermittent acute episodes of pain and flares have been observed in previous qualitative studies.^9,10,12,18^ Important similarities with these studies include the pain descriptors used (for example, sharp and stabbing), impact of the painful episodes, symptom variability, and pain associated with activity. Our study explored the impact of activity further and participants described the loss of confidence, feelings of vulnerability, and dependence on others owing to flares.

Our results echo the findings of Hawker *et al* ^10^ but additionally found that the term ‘flare’ could be used to describe both severe and minor events; however, the term was generally reserved for the former.

Predictability of episodes, which was variable, but generally associated with everyday activity, such as stair climbing, was important to people in our study as it meant they could plan ahead to participate in planned activities. Unpredictable episodes were more bothersome owing to the disappointment of having to cancel planned activities.

Murphy *et al* reported flares of variable intensity and duration.^18^ This was examined further and it was identified in the present study that the severe episodes of pain were more likely to be regarded as ‘flares’. The severe events described appear to be synonymous with the definition of a flare proposed by the Flare-in-OA OMERACT working group: *‘… it is a transient state, different from the usual state of the condition, with a duration of a few days, characterized by onset, worsening of pain, swelling, stiffness, impact on sleep, activity, functioning and psychological aspects, that can resolve spontaneously or lead to a need to adjust therapy.’* ^29^ This definition would not capture the minor events described in the present study, which may be a beneficial outcome for those wanting to study ‘flares’ or to differentiate between events causing more of an impact, reducing productivity, and leading to healthcare usage.

Help-seeking in the context of OA flares has not previously been explored but the findings are consistent with existing literature where people sought help owing to symptoms that had changed, lasted longer than normal, or were disruptive.^30,31^ The present study explored this further and people described seeking help when symptoms impacted on sleep, emotional limits were reached, self-management options exhausted, and pain experience was worse than normal. None of the participants sought help for minor events, which may in part be owing to them being perceived as ‘normal’. These minor events are likely to be largely ‘hidden’ from formal health care and not regarded as legitimate reasons for seeking help.

In tis study, recursivity (previous experience influencing future actions and help-seeking)^32^ shaped who participants thought would be most likely to help them or meet their expectations. Advice from peers had a significant impact on patients’ perceptions on management strategies and modified views on the need for knee replacement, which has previously been observed.^33^

### Implications for research and practice

The findings from this study highlight uncertainty about what flares are. The definition proposed by the Flare-in-OA OMERACT working group suggests only major episodes of pain are flares and this may resonate with many patients.^29^ A unified definition for a ‘flare’ would be helpful to ensure clinicians, people with OA, and researchers are using a common language to aid understanding in the healthcare setting and comparison of study findings in the research environment. Nevertheless, given the wide range of acute pain experiences, lay concepts, and language used, it should be expected that any such standardised definition will never perfectly capture these phenomena.

The importance of the more severe events are potentially more clear than minor events as they can lead to productivity loss and impact on daily living. It is still unclear whether minor episodes of pain represent a spectrum of flare severity or variability of OA symptoms. It is still undetermined whether distinguishing between these episodes of different severities is useful to people with OA, clinicians, or researchers. There is also uncertainty about how these episodes are prioritised by people with OA and clinicians in the context of comorbidities. However, it is important that people with OA are given the opportunity to seek help for episodes of acute pain, and they are supported to self-manage these by clinicians who understand that flares impact on wellbeing of people with OA and require help to deal with them.

Illustrating OA flares and pain experience diagrammatically may be a helpful tool within the primary care consultation about OA to understand frequency, severity, impact, and self-management strategies employed. Furthermore, ascertaining why people with OA seek help, which might encompass reaching emotional limitations, disturbed sleep, or pain lasting longer than usual or impacting on daily activity, is also important.
